# Supplementary material for: Comparative analysis of viral RNA signatures on different RIG-I-like receptors
Source: eLife. 2016 Mar 24;5:e11275. doi: 10.7554/eLife.11275 (PMC4841775; doi:10.7554/eLife.11275)
Supplement: Supplementary file 1. — DOI: http://dx.doi.org/10.7554/eLife.11275.019 [file elife-11275-supp1.pptx]

## Slide 1
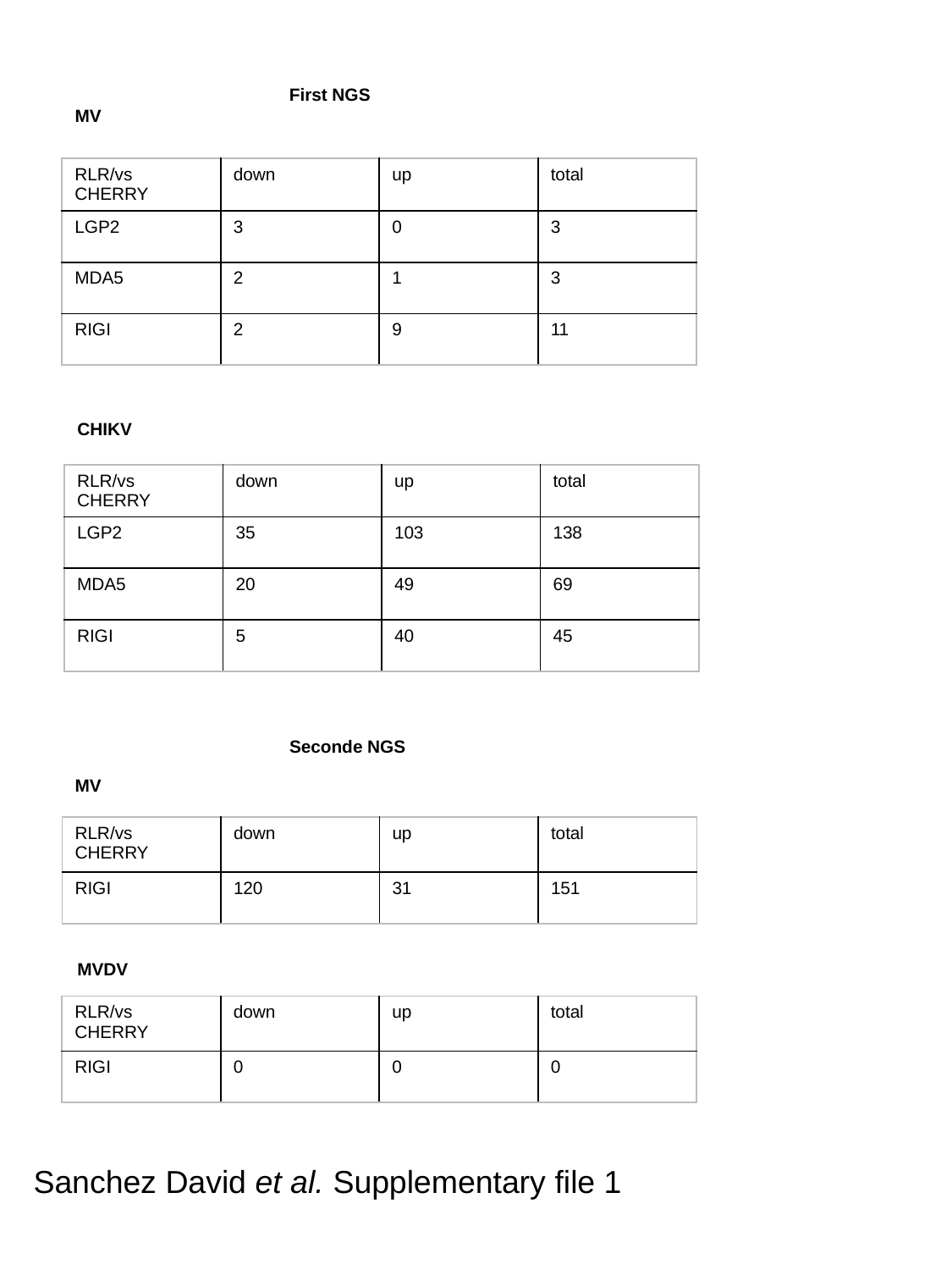

First NGS
MV
| RLR/vs CHERRY | down | up | total |
| --- | --- | --- | --- |
| LGP2 | 3 | 0 | 3 |
| MDA5 | 2 | 1 | 3 |
| RIGI | 2 | 9 | 11 |
CHIKV
| RLR/vs CHERRY | down | up | total |
| --- | --- | --- | --- |
| LGP2 | 35 | 103 | 138 |
| MDA5 | 20 | 49 | 69 |
| RIGI | 5 | 40 | 45 |
Seconde NGS
MV
| RLR/vs CHERRY | down | up | total |
| --- | --- | --- | --- |
| RIGI | 120 | 31 | 151 |
MVDV
| RLR/vs CHERRY | down | up | total |
| --- | --- | --- | --- |
| RIGI | 0 | 0 | 0 |
Sanchez David et al. Supplementary file 1
