# Supplementary material for: Comparative analysis of viral RNA signatures on different RIG-I-like receptors
Source: eLife. 2016 Mar 24;5:e11275. doi: 10.7554/eLife.11275 (PMC4841775; doi:10.7554/eLife.11275)
Supplement: Supplementary file 2. — DOI: http://dx.doi.org/10.7554/eLife.11275.020 [file elife-11275-supp2.pptx]

## Slide 1
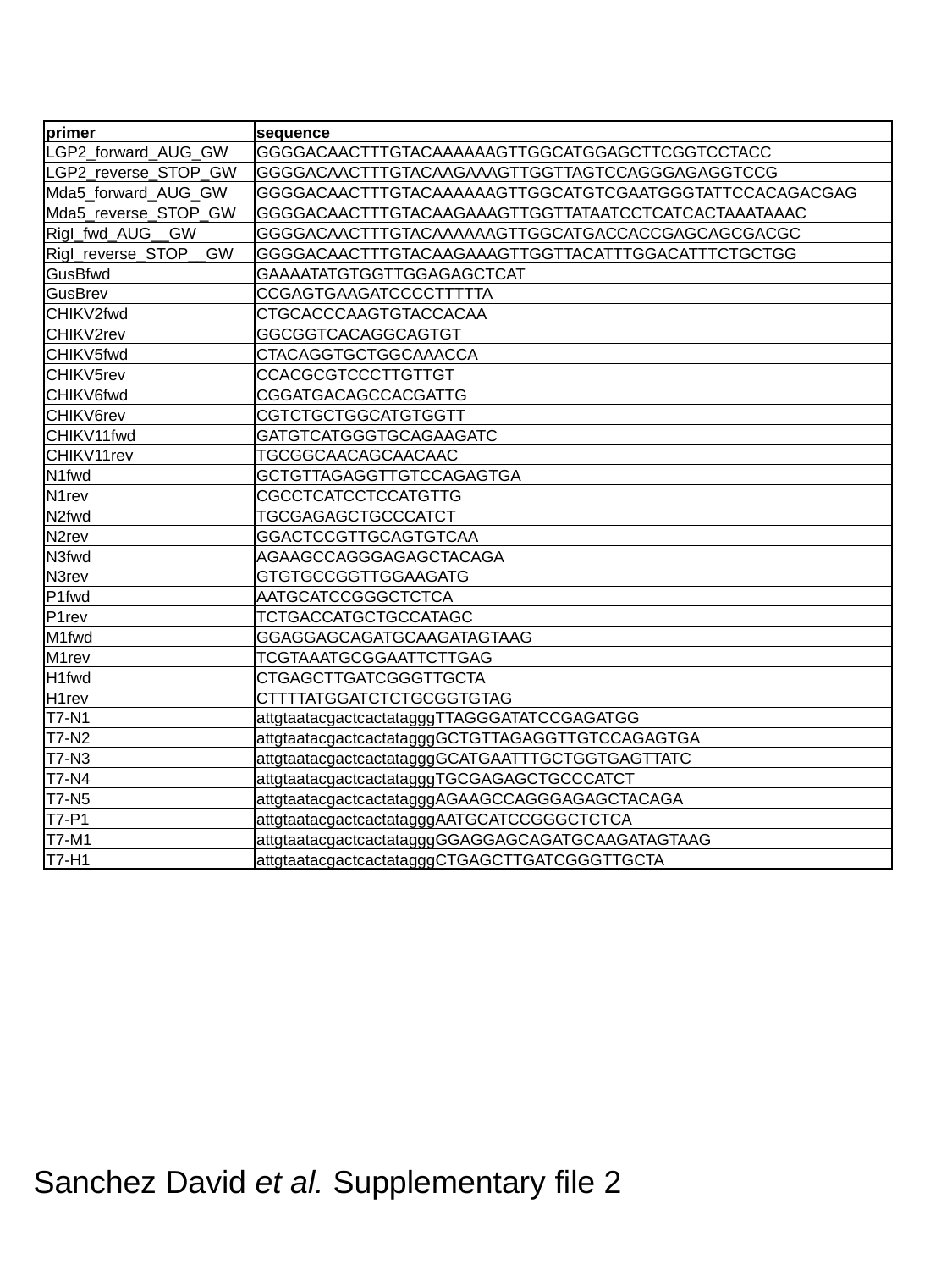

| primer | sequence |
| --- | --- |
| LGP2\_forward\_AUG\_GW | GGGGACAACTTTGTACAAAAAAGTTGGCATGGAGCTTCGGTCCTACC |
| LGP2\_reverse\_STOP\_GW | GGGGACAACTTTGTACAAGAAAGTTGGTTAGTCCAGGGAGAGGTCCG |
| Mda5\_forward\_AUG\_GW | GGGGACAACTTTGTACAAAAAAGTTGGCATGTCGAATGGGTATTCCACAGACGAG |
| Mda5\_reverse\_STOP\_GW | GGGGACAACTTTGTACAAGAAAGTTGGTTATAATCCTCATCACTAAATAAAC |
| RigI\_fwd\_AUG\_\_GW | GGGGACAACTTTGTACAAAAAAGTTGGCATGACCACCGAGCAGCGACGC |
| RigI\_reverse\_STOP\_\_GW | GGGGACAACTTTGTACAAGAAAGTTGGTTACATTTGGACATTTCTGCTGG |
| GusBfwd | GAAAATATGTGGTTGGAGAGCTCAT |
| GusBrev | CCGAGTGAAGATCCCCTTTTTA |
| CHIKV2fwd | CTGCACCCAAGTGTACCACAA |
| CHIKV2rev | GGCGGTCACAGGCAGTGT |
| CHIKV5fwd | CTACAGGTGCTGGCAAACCA |
| CHIKV5rev | CCACGCGTCCCTTGTTGT |
| CHIKV6fwd | CGGATGACAGCCACGATTG |
| CHIKV6rev | CGTCTGCTGGCATGTGGTT |
| CHIKV11fwd | GATGTCATGGGTGCAGAAGATC |
| CHIKV11rev | TGCGGCAACAGCAACAAC |
| N1fwd | GCTGTTAGAGGTTGTCCAGAGTGA |
| N1rev | CGCCTCATCCTCCATGTTG |
| N2fwd | TGCGAGAGCTGCCCATCT |
| N2rev | GGACTCCGTTGCAGTGTCAA |
| N3fwd | AGAAGCCAGGGAGAGCTACAGA |
| N3rev | GTGTGCCGGTTGGAAGATG |
| P1fwd | AATGCATCCGGGCTCTCA |
| P1rev | TCTGACCATGCTGCCATAGC |
| M1fwd | GGAGGAGCAGATGCAAGATAGTAAG |
| M1rev | TCGTAAATGCGGAATTCTTGAG |
| H1fwd | CTGAGCTTGATCGGGTTGCTA |
| H1rev | CTTTTATGGATCTCTGCGGTGTAG |
| T7-N1 | attgtaatacgactcactatagggTTAGGGATATCCGAGATGG |
| T7-N2 | attgtaatacgactcactatagggGCTGTTAGAGGTTGTCCAGAGTGA |
| T7-N3 | attgtaatacgactcactatagggGCATGAATTTGCTGGTGAGTTATC |
| T7-N4 | attgtaatacgactcactatagggTGCGAGAGCTGCCCATCT |
| T7-N5 | attgtaatacgactcactatagggAGAAGCCAGGGAGAGCTACAGA |
| T7-P1 | attgtaatacgactcactatagggAATGCATCCGGGCTCTCA |
| T7-M1 | attgtaatacgactcactatagggGGAGGAGCAGATGCAAGATAGTAAG |
| T7-H1 | attgtaatacgactcactatagggCTGAGCTTGATCGGGTTGCTA |
Sanchez David et al. Supplementary file 2
